# Further simulations of the effect of cochlear-implant pre-processing and head movement on interaural level differences

**DOI:** 10.1121/10.0005647

**Published:** 2021-07-01

**Authors:** Alan W. Archer-Boyd, Robert P. Carlyon

**Affiliations:** Cambridge Hearing Group, MRC Cognition and Brain Sciences Unit, University of Cambridge, 15 Chaucer Road, Cambridge CB2 7EF, United Kingdom

## Abstract

We simulated the effect of several automatic gain control (AGC) and AGC-like systems and head movement on the output levels, and resulting interaural level differences (ILDs) produced by bilateral cochlear-implant (CI) processors. The simulated AGC systems included unlinked AGCs with a range of parameter settings, linked AGCs, and two proprietary multi-channel systems used in contemporary CIs. The results show that over the range of values used clinically, the parameters that most strongly affect dynamic ILDs are the release time and compression ratio. Linking AGCs preserves ILDs at the expense of monaural level changes and, possibly, comfortable listening level. Multichannel AGCs can whiten output spectra, and/or distort the dynamic changes in ILD that occur during and after head movement. We propose that an unlinked compressor with a ratio of approximately 3:1 and a release time of 300–500 ms can preserve the shape of dynamic ILDs, without causing large spectral distortions or sacrificing listening comfort.

## I Introduction

Cochlear-implant (CI) listeners have an electric dynamic range of approximately 6–20 dB, which is considerably smaller than the 100-dB acoustic dynamic range of normally hearing (NH) listeners (Skinner *et al*., 1997; Zeng and Galvin, 1999; Hughes *et al*., 2001; Zeng *et al*., 2002; Theelen-van den Hoek *et al*., 2014). Automatic gain control (AGC) is used to compensate for this reduced dynamic range in CI listeners, and also for hearing-aid users where it is more commonly referred to as dynamic-range compression. In general, AGCs attempt to place most sounds in the environment within the limited dynamic range of the listener, while preventing exposure to loud sounds, especially those that are sudden. The AGCs currently used in CIs often apply the same compression across the entire frequency spectrum, and the compression can be completely characterised by a set of simple parameters such as the attack/release time, threshold, and compression ratio. These standard single-channel compressors are implemented in various configurations in Advanced Bionics (AB), Cochlear, and Med-El devices (Vaerenberg *et al*., 2014). Alternatively, AGCs can be multi-channel systems with very little in common with a standard compressor, except that their end goal, the preservation of audibility for the user, is the same. These “alternative” systems include adaptive dynamic range optimization (ADRO™) (Blamey *et al*., 2011), available as an option in Cochlear devices, and the transfer-function-based xDP™ and VoiceGuard™ systems implemented in Oticon devices (Bozorg-Grayeli *et al*., 2016; Segovia-Martinez *et al*., 2016).

The reduced spectral and temporal resolution of CI processing means that listeners are heavily dependent on temporal envelope cues to understand speech (e.g., Shannon *et al*., 1995; Shannon *et al*., 2001; Green *et al*., 2004). These cues can be distorted by fast-acting compressors (e.g., Stone and Moore, 2007). This means that slower-acting AGCs have previously yielded better speech intelligibility results in CI vocoder simulations (Stone and Moore, 2003), and has led to many CI manufacturers limiting fast-acting compression in AGCs to loud and/or transient sounds, with slow-acting compression or linear gain applied to all other sounds (Boyle *et al*., 2009).

A theoretically ideal hearing device would provide audibility for sounds that the listener wants to hear, maintain, or improve speech intelligibility in quiet and in noise, and preserve localization cues. However, there is an inevitable trade-off between these factors, and the parameters used in AGC systems are an important part of that trade-off. This is especially true in CIs, given listeners’ relatively narrow electrical dynamic range, and their much greater perceptual weighting of interaural level difference (ILD) cues over interaural time difference (ITD) cues for sound localization, relative to NH listeners, who generally weight low-frequency ITDs more than ILDs for broadband stimuli (e.g., Wightman and Kistler, 1992; Macpherson and Middlebrooks, 2002). ITD cues are generally unavailable to CI listeners because the pulses between bilateral processors in clinical use are not synchronized between the ears. Even under experimental conditions where ITD cues are preserved by presenting the cues directly to the processor, perception of those cues by CI listeners remains poor (Van Hoesel, 2004; Ihlefeld *et al*., 2015).

As we have shown previously (Archer-Boyd and Carlyon, 2019), slow-acting unlinked AGCs can strongly distort the ILD cues available to the listener. In particular, dynamic ILDs produced by head movements can interact with slow-acting AGCs to produce an “overshoot” effect, whereby ILDs continue to change after the head has stopped moving. Furthermore, the combination of a single-channel broadband AGC and a pre-emphasis high-pass filter produced low-frequency static ILDs that were of the opposite sign to those at high frequencies. This occurred because the AGC was driven by the more-intense high frequencies, where ILDs are originally high, but applied equally across the spectrum, including low frequencies where ILDs are naturally low. That previous paper implemented only one set of AGC parameters, based on the Advanced Bionics compressor (attack/release time = 240/1500 ms, compression ratio = 12:1). It neither addressed the contributions that changes to AGC parameters could have on dynamic output ILDs nor did it compare standard AGC systems to the alternatives available to CI listeners. Killan *et al*. (2019) showed a significant relationship between device manufacturer (AB, Med-El, and Cochlear Ltd) and localization error in a large cohort of bilaterally implanted children, and different AGC settings were highlighted as a possible driving factor in this relationship. Here, we investigate the effects of changes to the AGC parameters and functionality of standard AGC systems on the ILDs that are presented to CI listeners. We also examine the output of three alternative systems using similar inputs. In all cases, we consider the interaction between these systems and listener head movements relative to a fixed source. Our results will enable researchers, manufacturers, and users to predict how their choice of AGC and CI parameters will affect the trade-off between speech audibility, speech intelligibility, and the preservation of ILD cues in dynamic listening environments.

The present study first models the effect of head movement on the output of unlinked bilateral CI processors using a standard, single-channel AGC and parameter settings similar to those used in a range of clinical CI processors. It also models the effect of simply linking these AGCs. Simulations of three multi-channel alternatives to standard AGCs are described, and their response to level changes due to head movements are investigated. The paper is organized into several parts. The first part describes the single-channel AGC systems that have been simulated and the parameter settings chosen. Subsequent sections present the unilateral CI processor outputs and the broadband output ILDs that arise from simple level changes and from a number of simulated rotational head movements, and discuss the relationship between head movement and AGC systems and parameters. The second part describes a similar analysis applied to the multi-channel alternatives. Finally, we consider the limitations and implications of our simulations, and the extent to which the results of our parameter manipulations match some of the predictions given by Archer-Boyd and Carlyon (2019).

## II General Methods

### A Input creation and modeling head movement

The input to the AGC simulation was speech-shaped noise (SSN), and identical to that described by Archer-Boyd and Carlyon (2019), and was generated in the same way. The frequency spectrum of the noise was created using the average spectrum of 30 Institute of Electrical and Electronic Engineers (IEEE) sentences from eight speakers (four male) of British English (Stacey and Summerfield, 2007). Our recording consisted of 320 sentences, having a total audio duration of 9 min 35 s. A new segment of SSN was generated for each head movement shown by applying random phases to the magnitude of each frequency component in the spectrum of the concatenated speech, then applying an inverse Fourier transform to produce SSN. This technique was similar to that found in the “Oscillator and Signal Generator” available on the Mathworks file exchange (Brimijoin, 2012). The segments varied in length due to the different durations of head movement used, but all included 10 s before the head movement at the starting position of the head, allowing the compressor to stabilize Head movements were simulated using an offline “overlap-add” method described in full by Archer-Boyd and Carlyon (2019), and based on the real-time method described by Brimijoin *et al*. (2013). As a substitution for a CI microphone, an impulse-response library of behind-the-ear hearing-aid microphone responses was used (Kayser *et al*., 2009). The library used Siemens Acuris hearing aids mounted on a KEMAR head-and-torso simulator. Recordings were made at 5° intervals at a distance of 3 meters in an anechoic chamber. The head movement simulated here was sinusoidal; this is more similar to the natural movement of the head than was the case for the linear, constant-speed head movement simulated by Archer-Boyd and Carlyon (2019).

### B Output treatment

The output of each filter channel was smoothed by calculating the root mean square (RMS) value over a sliding 50 ms rectangular window. This was achieved using the “envelope” function in MATLAB. Smoothing served to make overall level changes over time clearer in the plots. No additional vocoding step nor additional envelope extraction were included in the signal path. For broadband plots, the outputs of the channels were recombined.

### C Summary metrics

**Fig. 1:**
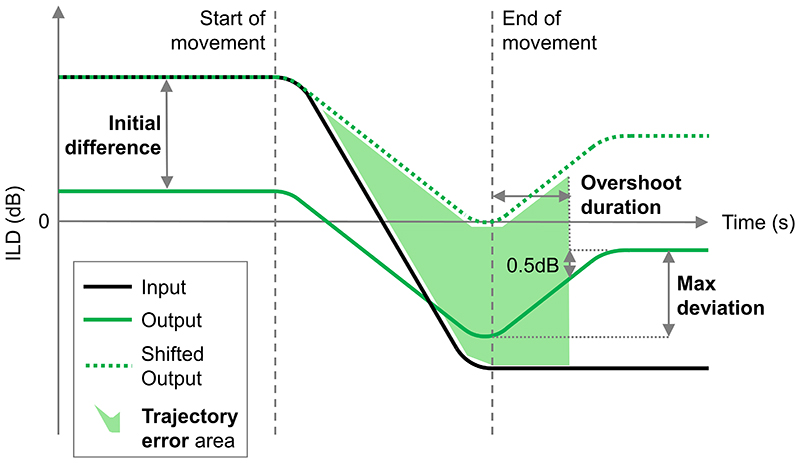
(Color online) Annotated diagram showing how the summary metrics are defined. The summary metrics are in bold.

In order to compare across settings and systems, a number of summary metrics have been calculated. These metrics are shown graphically in Fig. 1. The “overshoot duration” is the amount of time taken for broadband output ILD to be within 0.5 dB of its final value (calculated using the final second of the output) after the head has stopped moving. The “initial difference” is the mean difference between the input and output ILDs in the second before head movement starts. The “trajectory error” is the root-mean-squared error (RMSE) between the input and output ILDs between the start of the head movement and the end of the overshoot (the “trajectory error area” shaded in green in Fig. 1). The initial difference is added to the output ILD (“Shifted Output” in Fig. 1) before calculation of the trajectory error in order to better track differences in trajectory rather than absolute differences in ILD. Finally, the “max deviation” is the difference between the minimum output ILD value (the lowest, signed output ILD value), and the final ILD value. This provides another measure of the amount of ILD trajectory distortion. When mean values are quoted in the text, they will be given with their standard deviation in parentheses.

## III Standard AGC Simulation

**Fig. 2:**
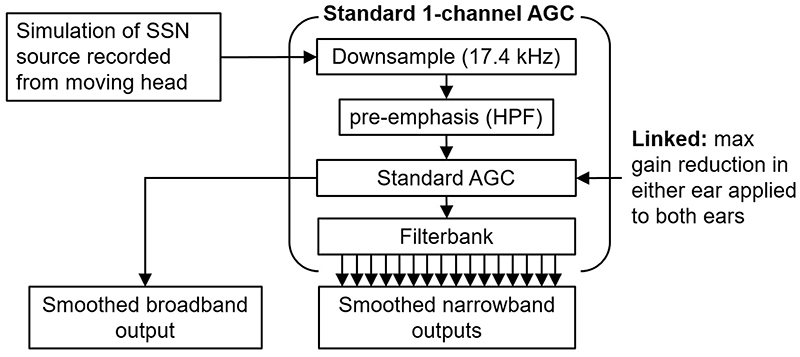
Schematic showing the signal path for a standard, single-channel CI AGC simulator. “HPF” stands for high-pass filter.

The simulation of a CI signal path with a standard AGC—up to the output of the filterbank—is shown in Fig. 2. The sample rate was 17.4 kHz. The simulated audio from a moving head and static sound source was used as the input to a high-pass pre-emphasis filter identical to the one used by Archer-Boyd and Carlyon (2019) and described in detail by Boyle *et al*. (2009). A makeup gain of 4.6 dB was applied to the output to approximately equalize the signal level before and after filtering. This formed the input to the AGC.

The output of the AGC was filtered using a bank of 16 sixth-order Butterworth bandpass filters (3rd order each side) with the same spacing and bandwidth as used in the Advanced Bionics Cochlear Implant (AB CI) described in Archer-Boyd and Carlyon (2019). We acknowledge that other CI processors have different channel bandwidths, spacing, number of channels, and types of filterbanks. These parameters were kept constant here so as to make the effect of changing individual AGC parameters clearer, and with respect to the filterbank, easier to reproduce. In the linked AGC simulation, a simple max gain reduction rule was used that applied the max gain reduction calculated at either compressor to both the left and right inputs.

A standard AGC system or dynamic-range compressor consists of a level detector (root-mean-square, rms, or peak, either in the linear or logarithmic domain) that produces a smooth control signal that tracks the envelope of the input. The degree of smoothing is controlled by time constants: an attack time constant to track increases in input level, and a release time constant to track decreases in level. The gain reduction applied to the input is calculated from the level input/output function of the compressor, which has two parameters: threshold (the control signal level above which compression is applied), and compression ratio (the ratio between input and output levels above threshold). The AGC envelope detector used here can be defined as a one-pole filter in the digital domain (based on Giannoulis *et al*., 2012),

(1) $$d[n]=\{\begin{array}{ll} \alpha d[n-1]+(1-\alpha )x[n], & \left|x[n]|\geq d[n-1\right]\\ \beta d[n-1]+(1-\beta )x[n], & |x[n]|<d[n-1], \end{array}$$

where *x*[*n*] is the rms (in dB) of the input for a single audio frame (256 samples), *n* is a time step, *d*[*n*] is the envelope detector output, *α* is the attack-time constant, and *β* is the release-time constant. *α* and *β* are defined by Giannoulis *et al*. (2012), from the step response of the filter, as

$$\alpha =e^{-M_{step}/\tau_{a}f_{s}},$$,

$$\beta =e^{-M_{step\;}/\tau_{r}f_{s}},$$,

where *M_step_* is the step size in samples for each audio frame, *τ_a_* and *τ_r_* are the attack and release times in seconds, and *f_s_* is the sample rate.

A gain reduction is applied to the signal when the envelope tracker is above a threshold level, normally defined in dB, according to the compression ratio of input/output signals. For example, if the envelope signal d[n] is 64 dB sound pressure level (SPL), 4 dB above the threshold level of 60 dB SPL, and the compression ratio is 4:1, then the input level is 4 dB above threshold, and the output level should be 1 dB above threshold. Therefore, a gain reduction of 3 dB will be applied to the input to produce the desired output signal level for the compression ratio. The compression algorithm used in this study uses a so-called “hard” knee. This means that the compressive input-output function resembles a broken stick, and the compression ratio is applied fully when the envelope tracker is above threshold (in contrast to a “soft” knee, whereby the input-output function is curved at around the threshold value). The envelope signal may be calculated using the peak input level or the rms. Here, the rms value of each audio frame was used, as AGC thresholds, inputs, and outputs are often quoted in dB SPL, which is itself an rms measure. We define threshold level as the level at which the compressor starts to act (compression ratio >1:1). Note that Moore (2008) (after ANSI, 2003) defines threshold as being “the input level at which gain is reduced by 2 dB, relative to the gain applied in the region of linear amplification.” This is in many ways a more difficult parameter to use, as it varies with compression ratio. We, therefore, have not used this definition in the simulations.

In order to avoid any confusion over the reporting of the attack and release times, we provide both the ANSI S3.22 (ANSI, 2003) defined measure of the attack and release times (Table I), and the arguably more standard and descriptive attack and release times used to calculate the time constants, see also Stone *et al*. (1999). ANSI attack and release time measurements were made here using a complex of 2- and 4-kHz sine tones. The rms level of each tone was −45 dBFS (dB re full scale) for 5 s, then instantaneously stepped to −10 dB for 5 s, and returned to −45 dBFS for 5 s. Zero dBFS was nominally set at 100 dB peak level for a sine tone used in some CIs, allowing input levels of up 97 dB SPL to be simulated without clipping occurring. Therefore, the input increased from 55 to 90 dB SPL and back again, as specified in the ANSI standard (ANSI, 2003). For clarity and to aid reproducibility, the ANSI measurements were made using only the AGC algorithm (i.e., no microphone impulse responses were used, and no pre-emphasis filtering or makeup gain was applied). Table I shows the ANSI-defined attack and release times, and compression ratios used in the subsequent simulations, and the attack and release time constants. The parameters were chosen to cover the approximate range of values given in the literature for CI processors that include standard AGCs (Vaerenberg *et al*., 2014). The fast-acting part of the dual-loop AGCs used in CIs that utilize the dual-loop AGC (e.g., Advanced Bionics and Med-El) has not been implemented here; however, the inputs do not contain any transients that would trigger a response from the fast-acting part. The linked AGC implemented here is exactly the same as the unlinked AGC, except that the max gain reduction calculated at either ear is applied to the input at both ears (Fig. 2).

## IV Standard AGC Results

### A Unlinked standard AGC and inputs with a simple level change

In order to determine the effects of AGC parameter changes on a simple change in level, the simulator was presented with non-spatialized SSN signals. These signals changed linearly by ±6dB in level at a rate of ±6dBs^−1^ at a starting level of 60 dB SPL for increasing levels, and 66 dB SPL for decreasing levels. AGC threshold was set at 60 dB SPL/–40 dBFS, and pre-emphasis filtering was applied. The stimulus duration was 4 s and the level changes occurred over 1-s starting 1 s after stimulus onset.

**Fig. 3:**
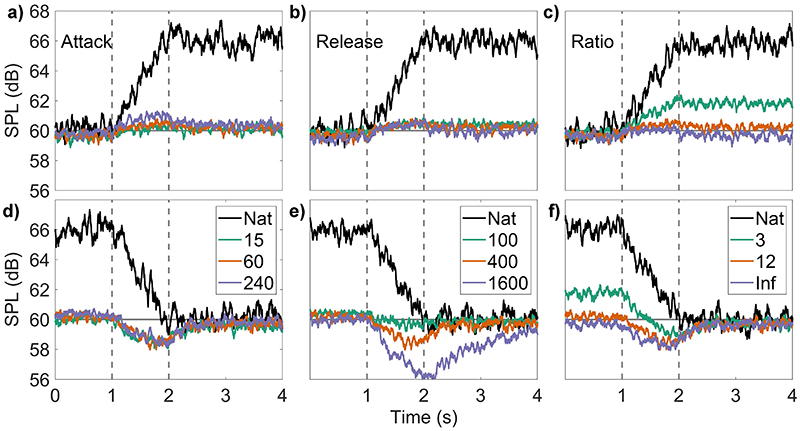
(Color online) Input and output level changes for +6dBs^−1^ (top row, a–c) and −6 dBs^−1^ (bottom row, d–f) input level change. Nat, natural input (no AGC, no pre-emphasis filter). The top row (a–c) shows increasing level, and the bottom row (d–f) shows decreasing level. The left column (a and d) shows changes to the attack time, the middle column (b and e) shows changes to the release time, and the right column (c and f) shows changes to the compression ratio. Unless otherwise stated, the AGC parameters are attack, 60 ms (ANSI); release, 400 ms (ANSI); ratio, 12. Threshold, 60 dB SPL. The colors denote low to high values in each, in the order green, orange, purple. The areas of the plots bounded by the dashed lines show the region of level change.

The results are shown in Fig. 3, with the input level changes shown in black and the start and end points of those changes shown by the vertical dashed lines. The columns left to right show the effect of changing the attack time, release time, and compression ratio, respectively, with the different parameters indicated by different colors within each plot. The baseline parameter values in each plot are attack time 60 ms, release time 400 ms, and compression ratio 12:1. All parameters used cover a similar range of times to those found in clinical devices. The top row [Figs. 3(a)–3(c)] shows the output for increasing levels. These outputs show very small (≤1 dB) changes due to changing the attack time [Fig. 3(a)], as indicated by the separation between the different-colored lines, but not by changes in the release time [Fig. 3(b)]. Conversely, inputs decreasing in level [bottom row, Figs. 3(d)–3(f)] are affected by changes in the release time [Fig. 3(e)], but not the attack time [Fig. 3(d)]. Both increasing- and decreasing-level sounds are affected by changes to the compression ratio [right column, Figs. 3(c) and 3(f)]. The differences between the outputs are much smaller for the attack time changes than for the release time changes. The time delay between the end of a level change and the output level stabilizing is referred to as the “overshoot.” Overshoot is a continued change in level after the input level to one ear has stopped changing; i.e., at the time point indicated by the rightmost vertical dashed line in each plot. In Fig. 3, overshoot is much more pronounced for level decreases than increases, and the duration of the overshoot increases most clearly with increasing release time. For example, with a decreasing level and a 1600 ms release time [Fig. 3(e)], the AGC produces a level reduction of up to 4 dB for 2 s after the input level has stopped changing (from 2 to 4 s). The next section shows that the overshoot in these level changes, shown here for a monaural input, will produce corresponding changes in ILD.

### B. Unlinked AGC and head movement

Simple level changes can be useful for investigating the effect of AGC on level; however, they neglect the effect of the position of each processor at the left and right ears, and the movement of the head. In the following, a sound source was simulated at 0° in front of a listener at a distance of 3 meters in an anechoic room, using the front microphones of ear-mounted behind-the-ear (BTE) hearing aids as the input. Simulated head movement followed a sinusoidal trajectory. The trajectory starts at −60°, with the head pointing to the left of the source, and ends 2 s later at +60°, pointing to the right. We use the same trajectory direction throughout the simulations presented in this article.

**Fig. 4:**
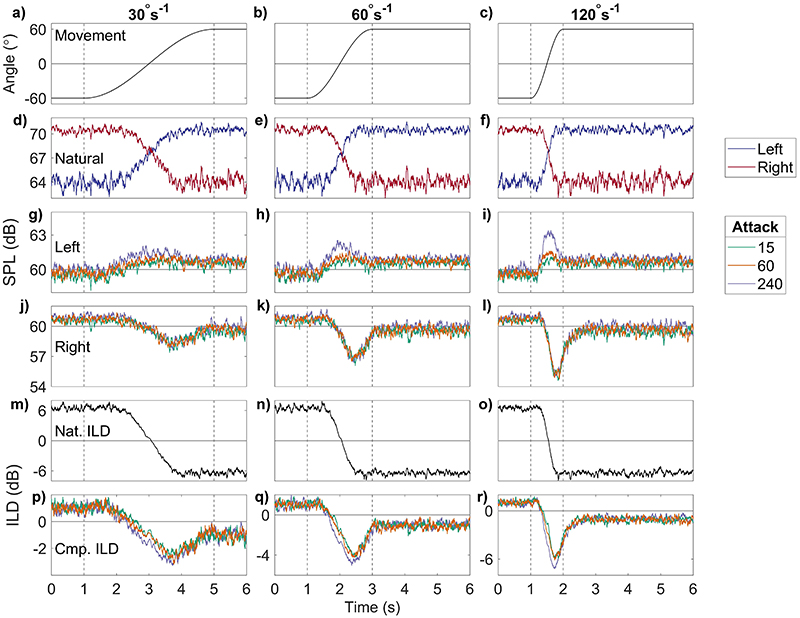
(Color online) Head movements, left and right input and output levels for three attack times, and input and output ILDs plots, for a SSN input at 64dB SPL (left ear). Each column displays a different rotational velocity. The left column (a, d, g, j, m, p) shows 30°s^−1^, the middle column (b, e, h, k, n, q) 60°s^−1^, and the right column (c, f, i, l, o, r) 120°s^−1^. The top row (a–c) shows head movement, second from top row (d–f) shows the input levels at both ears, third from top row (g–i) shows the output levels of simulations at the left ear, third from bottom row (j–l) shows the same for the right ear, second from bottom row (m–o) shows the input ILDs, and the bottom row (p–r) shows the output ILDs. The colors denote low to high values in each, in the order green (15 ms), orange (60 ms), purple (240 ms). The areas of the plots bounded by the dashed lines show the duration of rotational movement. Constant AGC parameters are release, 400 ms (ANSI); ratio, 12; threshold, 60 dB SPL.

Figure 4 shows the results of using AGC attack times of 15 (green), 60 (orange), and 240 (purple) ms. In all conditions, the release time was 400 ms and the compression ratio was 12:1. The top row [Figs. 4(a)–4(c)] shows the simulated head movement that produced the input, output, and ILD plots in the rows below. The second from the top row [Figs. 4(d)–4(f)] shows the input broadband levels at both ears; the third from the top row [Figs. 4(g)–4(i)] shows the output levels for the left ear; the third from bottom row [Figs. 4(j)–4(l)] shows the output levels for the right ear; the second from the bottom row [Figs. 4(m)–4(o)] shows the input broadband ILD; and the bottom row [Figs. 4(p)–4(r)] shows output broadband ILD. Head rotational velocity increases across columns from left to right. The left column [Figs. 4(a), 4(d), 4(g), 4(j), 4(m), and 4(p)] shows the results for 30°s^−1^, right column [Figs. 4(b), 4(e), 4(h), 4(k), 4(n), and 4(q)] 60°s^−1^, and right column [Figs. 4(c), 4(f), 4(i), 4(l), 4(o), and 4(r)] 120°s^−1^, such that the level change occurs over 4, 2, and 1 s respectively.

The different attack times affect the AGC output in the left ear [third from top row, Figs. 4(g)–4(i)], where the head movement causes the level to increase, but not in the right ear (third from bottom row, Figs. 4(j)–4(l)], where the levels decrease. Slow movement [Fig. 4(g)] results in very little difference (maximum 1 dB) between the left-ear outputs for each attack time. At 60°s^−1^ [Fig. 4(h)], the max difference between the shortest and longest attack times is 1.9 dB. At 120°s^−1^ [Fig. 4(i)], the max difference increases to 2.4dB. At this rotational velocity, output level also remains constant for a portion of the movement (duration 0.35 s, from 1.4 to 1.75 s). As the right ear also changes in level, the maximum difference in ILD between attack times was slightly smaller than the maximum difference in left-ear level; it was approximately 1 and 1.5 dB at 60°s^−1^ [Fig. 4(q)] and 120°s^−1^ [Fig. 4(r)], respectively. The bottom row [Figs. 4(p)–4(r)] shows that across all velocities, the small differences in ILD (≤1dB) produced by different attack times is largest in the first half of the movement, and converges to the same trajectory in the second half of the movement.

The summary ILD metrics are shown in Table II. They show overshoot only at 120°s^−1^ of duration 0.26 to 0.29 s, increasing with attack time. The initial difference between the input and output ILDs was 5.38(7) dB on average. Trajectory error was largest at 30°s^−1^ at 6.3(1) dB, 5.4(2) dB at 60°s^−1^, and 5.6(2) dB at 120°s^−1^. Max deviation increased with attack time and movement speed, from 1.8 to 6.3 dB as attack time increased from 15 to 240 ms and movement speed from 30 to 120°s^−1^. These differences are very small and may not be perceptually relevant. The most obvious change apparent in these simulations is that the output level at each ear changes non-monotonically despite a monotonically changing input level, which in turn results in a non-monotonically changing ILD.

**Fig. 5:**
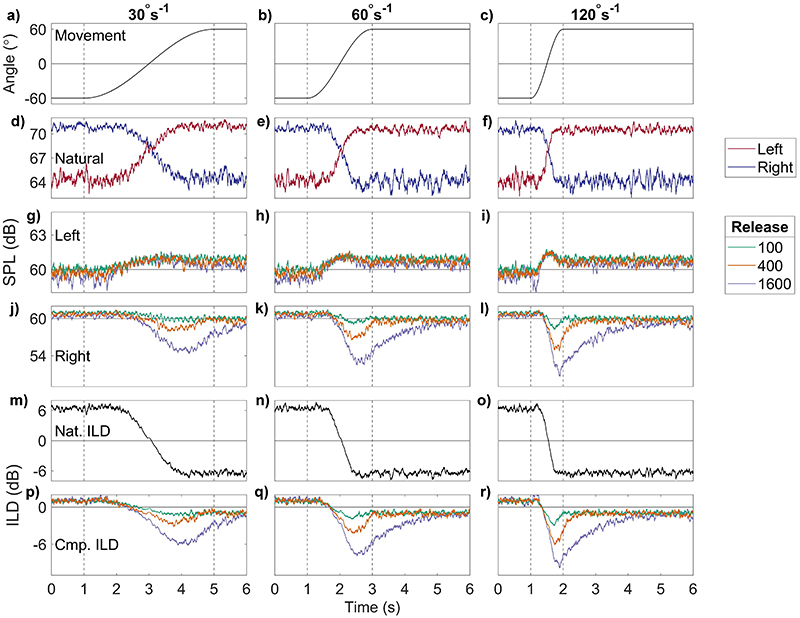
(Color online) The same as Fig. 4, for changing release times: green (100 ms), orange (400 ms), purple (1600 ms). Constant AGC parameters are attack, 60 ms (ANSI); ratio, 12; and threshold, 60 dB SPL.

Figure 5 shows the results of using AGC release times of 100 (green), 400 (orange), and 1600 (purple) ms. Attack time was 60 ms, and the compression ratio was 12:1, for rotational velocities of 30, 60, and 120°s^−^^1^. The layout of the figure is identical to Fig. 4. Release time affects the outputs only for the right ear (where the level decreases), unlike the case for attack times. Slow movement [left column, Figs. 5(a), 5(d), 5(g), 5(j), 5(m), and 5(p)] produces large differences between the outputs for each release time [Fig. 5(p)], and these increase further for faster movements [Figs. 5(q) and 5(r)]. A release time of 100 ms (green) results in<1 dB change in level, 400 ms (orange) causes a change of 2dB, and 1600ms (purple) 5dB. At 60°s^−1^ [Fig. 5(q)], the maximum level changes for release times 100, 400, and 1600 ms are <1, 3.3, and 7.5 dB respectively, and at 120°s^−1^ [Fig. 5(r)], the maximum changes are 1.4, 5.3, and 9.4 dB.

The summary ILD metrics are shown in Table III. The metrics show non-zero overshoot durations for 1600 ms release time across all head movements from 0.27 s at 30°s^−^^1^, to 1.52 s at 60°s^−^^1^, and 1.62 s at 120°s^−^^1^. At 120°s^−^^1^, an overshoot duration of 0.28 s was also measured at 400 ms release time. The initial difference between the input and output ILDs was 5.4(1) dB on average. Trajectory error was largest (7 dB) at the shortest release time (100 ms) and slowest speed (30°s^−^^1^). The lowest trajectory error was 5 dB (1600 ms, 30°s^−^^1^). There was no clear pattern to these results across conditions. The max deviation showed a clearer relationship to parameter changes, increasing with release time and movement speed, from 0.7 dB at release time = 100 ms and 30°s^−^^1^, to 8.6 dB at release time = 1600 ms and 120°s^−^^1^.

**Fig. 6:**
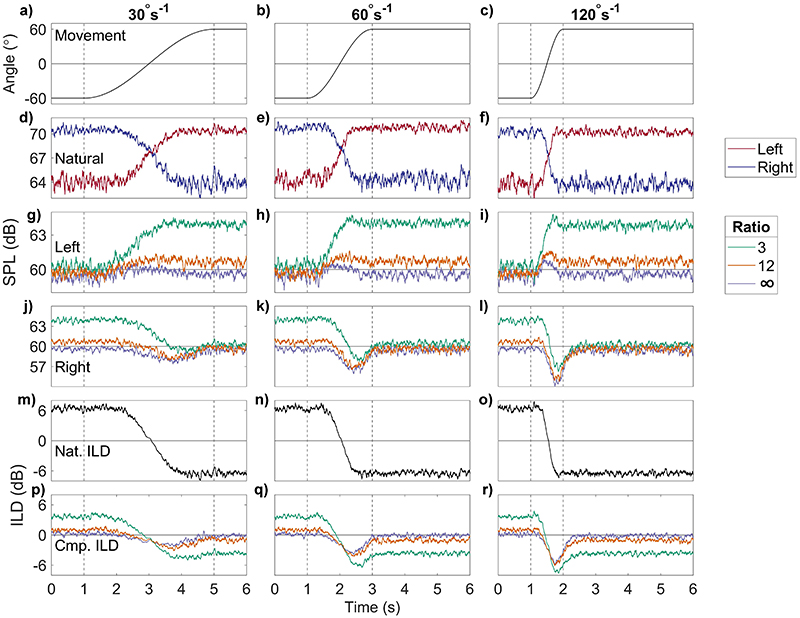
(Color online) The same as Fig. 4, for changing compression ratios: green (3:1), orange (12:1), purple (∞:1). Constant AGC parameters are attack, 60 ms; release, 400 ms (ANSI); and threshold, 60dB SPL.

Figure 6 shows the results of using AGC compression ratios of 3 (green), 12 (orange), and ∞ (purple) ms. Attack time was 60 ms, and the release time was 400 ms, for rotational velocities of 30, 60, and 120°s^−1^. Increasing compression ratio decreases output levels that are higher than the AGC threshold of 60 dB [middle rows, Figs. 6(g)–6(l)]. Changing the compression ratio affects both the left and right output levels. The left-ear levels [third from top row, Figs. 6(g)–6(i)] increase monotonically from 60 dB to 61 and 64 dB at compression ratios of 12:1 and 3:1, respectively, and show smaller and sometimes non-monotonic changes at a ratio of ∞:1. Levels at the right ear (third from bottom row, Figs. 6(j)–6(l)] first decrease then increase during head movement, continuing to change after head movement has stopped and resulting in clear overshoot in the 120°s^−1^ condition [Fig. 6(r)].

The summary ILD metrics are shown in Table IV. The metrics show non-zero overshoot durations at 120°s^−1^ at all compression ratios [0.25(2) s]. The initial difference between the input and output ILDs increased with increasing compression ratio, from 2.8 dB at 3:1, to 5.4 dB at 12:1, and 6.4 dB at ∞:1 (standard deviation across movement velocity was negligible and due to fluctuations in the noise input). Trajectory error increased with increasing compression ratio and decreased with increasing velocity, from 2.2 dB at 3:1 and 120°s^−1^ to 7.4 dB at ∞:1 and 30°s^−1^. Standard deviation across velocities for each compression ratio was 0.5–0.6 dB. Max deviation increased with compression ratio and movement speed, from 1.4 dB at 3:1 and 30°s^−1^, to 5.8 dB at ∞:1 and 120°s^−1^. ILDs are not more than 1.5 dB different between the 12:1 and ∞:1 conditions across all rotational velocities. The slowest rotational velocity and lowest compression ratio result in an ILD and level change most similar to the natural input changes.

The effect of the high-pass pre-emphasis filter has not been shown as an explicit manipulation and deserves some discussion. The pre-emphasis filter alters the spectrum of the input before compression. When combined with the fixed makeup gain applied to both signals after pre-emphasis, this results in a level reduction at the contralateral ear due to the head shadow, and a level increase at the ipsilateral ear due to a lack of head shadow. As the pre-emphasis filtering increases the relative weighting of the high frequencies, which have larger ILDs, in the energy of the broadband signal, this results in larger broadband ILDs before compression. The ILDs in each frequency band remain unaffected by the pre-emphasis filter.

Isolating and altering each parameter of the pre-AGC and unlinked AGC processing raises some key points. Both the simple level change and head movement results show that sounds increasing in level are affected only by the attack time, and sounds decreasing in level are affected only by the release time. The small range of short attack times used in clinical devices means that there is very little difference between the outputs of the attack times used, except at the highest rotational velocity. Short attack times combined with high compression ratios greatly attenuate the increases in level during head turns. The longer and wider range of release times used here and across clinical devices result in level changes comparable to the natural input changes, as shown in the bottom row of Fig. 5. Overshoot occurs with longer release times and/or faster head turns, and lower compression ratios only partially mitigate this. Together these results suggest that the best parameters to preserve the profile of ILD changes during head movement are a low compression ratio, a short- to mid-duration release time, and attack times that are perhaps longer than those currently used in standard CI AGCs. Currently, the slow-acting part of Med-El’s AGC parameters are closest to this ideal (attack, 100ms; release, 400 ms; compression ratio:, 3:1, variable threshold). Plots using the parameters closest to Med-El’s are shown by the green lines in Fig. 5 (i.e., attack, 60 ms; release, 400 ms; compression ratio, 3:1). The Advanced Bionics parameters are attack, 240 ms; release, 1500 ms; compression ratio, 12:1; and the Cochlear Device’s AGC (not the additional ADRO™ processing option) uses attack, 5 ms; release, 65 ms; compression ratio, ∞:1 (Vaerenberg *et al*., 2014).

The above results are for the unlinked AGCs, which is the configuration currently implemented in clinical use. Linking AGCs has also been proposed as a method to preserve ILDs, and we examine the output of a simple “max gain reduction” linked AGC system in Sec. IV C.

### C Simple linked AGC and head movement

**Fig. 7:**
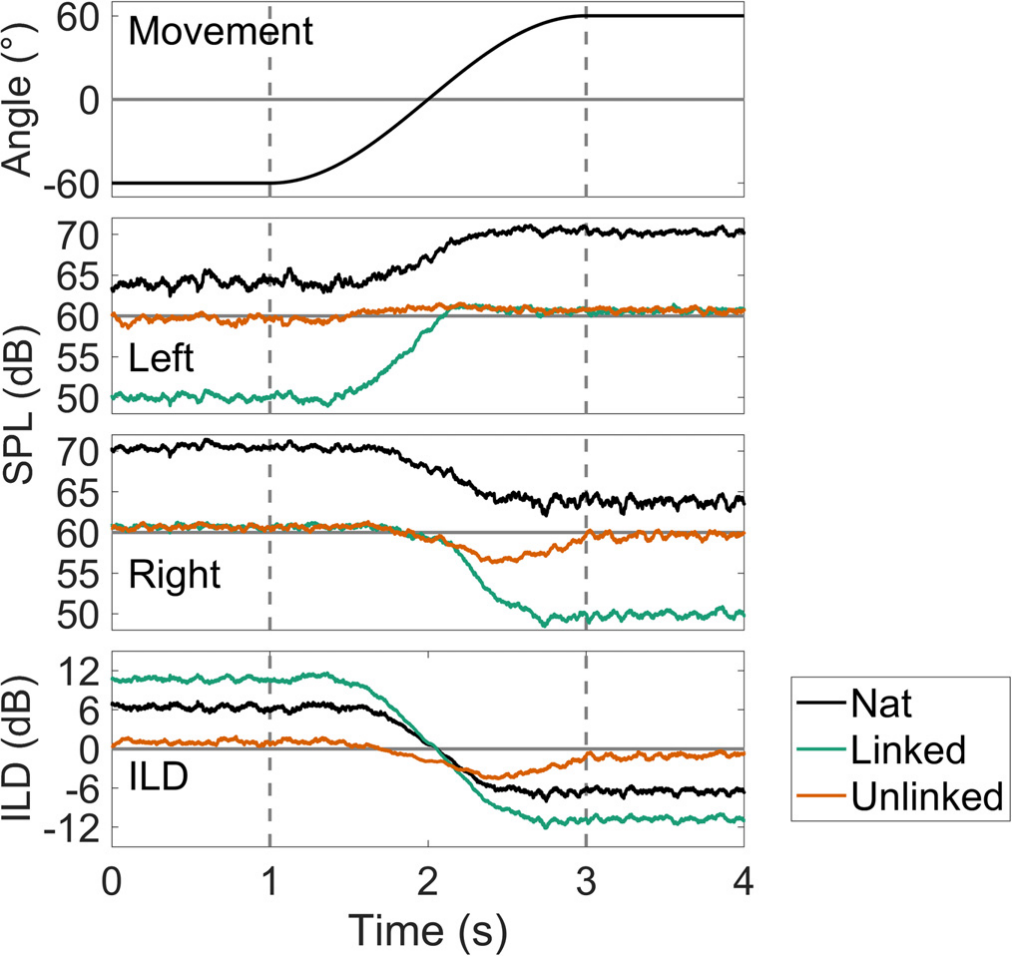
(Color online) Head movement, left and right natural (black) and max gain reduction linked (green) and unlinked (orange) compression levels, and natural, max gain reduction linked and unlinked compression ILD plots, for a SSN input at 64dB SPL (left ear). Rotational velocity is 60°s^−1^ from −60° to 60°. The areas of the plots bounded by the dashed lines show the duration of rotational movement.

Figure 7 shows the natural input levels, simple linked AGC output levels, and the corresponding ILDs for the input and output levels. Attack/release times were 60/400 ms, threshold was 60 dB SPL, compression ratio was 12:1, and pre-emphasis filtering was applied. Rotational velocity was 60°s^−1^ for 2 s. The top plot shows the simulated head movement, the second and third plots show the input (black) and output (green) level at the left and right ears, and the bottom plot shows the input and output ILDs.

The natural input level change (black lines, second and third plot) is ±6dB, which results in a −12 dB change in broadband ILD (black line, bottom plot). The pre-emphasis filtering, combined with the linked AGCs results in a larger change in level of ±10.5 dB. The level in the right ear drops monotonically before reaching a steady state, rather than, as in most cases in Figs. 4 and 6, increasing and then decreasing. The *broadband* ILD change is 22 dB, almost double the input ILD change of 12 dB. The output ILD change, though much larger than the input ILD, is very similar in shape to the input, and no overshoot is observed. The summary metrics are shown in Table V. Trajectory error is similar for both the linked and unlinked systems (5.2 and 5.4 dB, respectively). However, initial difference was –4.3 dB for the linked system and 5.4 dB for the unlinked, reflecting the high-frequency pre-emphasis filter effects on broadband ILD. Max deviation was reduced by the linked compression from 3.6 to 1.4dB. Linked AGC results in minimal monaural level reversals and overshoot. However, combined with the effects of the pre-emphasis filter, the linked AGC produces large level changes that are approximately a sixth of the input dynamic range of the CI listener (typically 60 dB in AB and Med-El devices, less in Cochlear devices, and greater in Oticon devices) (Vaerenberg *et al*., 2014), and could result in an unnaturally large ILD being applied to an input at the expense of audibility in the contralateral ear.

## V Adaptive Dynamic Range Optimization Simulation and Results

### A ADRO™ simulation

**Fig. 8:**
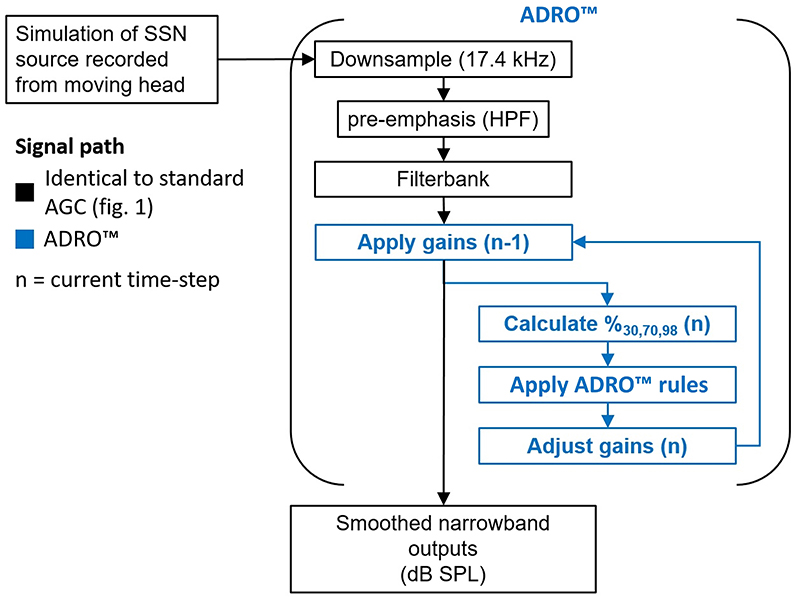
(Color online) Schematic showing the signal path for the ADRO™ simulator.

ADRO™ is a multi-channel system that aims to present most of the sounds a listener wishes to hear in each frequency band within the listener’s dynamic range of hearing, without compressing the sound. It does this by using multichannel, adaptive linear amplification. The signal path of the full ADRO™ system is shown in blue in Fig. 8. The input signal is statistically analysed to find the most information-rich section of each frequency band. Gain is controlled using fuzzy logic to maintain the level of that part of the sound within the audible range of the listener. The system uses five processing rules, applied independently in each frequency channel. The comfort rule reduces the gain if the channel output level exceeds a comfort target level more than 2% of the time. The background noise rule reduces the gain if the channel output level exceeds a specified background level more than 40% of the time. The audibility rule increases gain if the channel output level falls below an audibility target level more than 70% of the time. The hearing protection rule limits the maximum output level of a channel, and a further max gain rule limits the amount of amplification that can be applied, which stops very quiet sounds from being over-amplified.

The exact operation of the ADRO™ system can be found in the patent of Blamey *et al*. (2011), and the simulation used here is based on that patent and the values used in James *et al*. (2002). Three percentiles in each frequency channel are calculated from the long-term output level for each frequency band. These percentiles are used as targets for three of the five rules described above. In James *et al*. (2002), the 98th percentile was used for the comfort rule, 70th percentile for the audibility rule, and 40th for the background noise rule. These are mapped to three values for each frequency channel based on the listener’s dynamic range. Here, the 98th percentile was mapped to 75 dB SPL in every channel, 70th to 60 dB SPL, and 40th percentile to 45 dB SPL. This 30-dB dynamic range was the same as used by James *et al*. (2002) but mapped to a higher level for ease of comparison with the other AGCs modelled. In James *et al*. (2002), the rate of change of the percentile estimator was set at a relatively fast 20 dBs^−1^. The exact value used in the clinical Cochlear device is proprietary but is assumed to be similar. The gain change rate can also be set. Again, this is proprietary for the clinical device, but given as ±6 dBs^−1^ in James *et al*. (2002). It has also been quoted as 3 dBs^−1^ for increases and 9 dBs^−1^ for decreases in gain (Blamey, 2005; Moore, 2008). In total, ADRO™ has eight main adjustable parameters per frequency band: three percentiles, three target SPLs, a percentile estimator rate of change, and a gain rate of change. When ADRO™ is applied in CIs, the output levels are defined in terms of electrical dynamic range. For ease of comparison with the standard AGC, and to avoid the added complications of the different mapping laws between manufacturers, the output will be given in dB SPL.

### B ADRO™ results

**Fig. 9:**
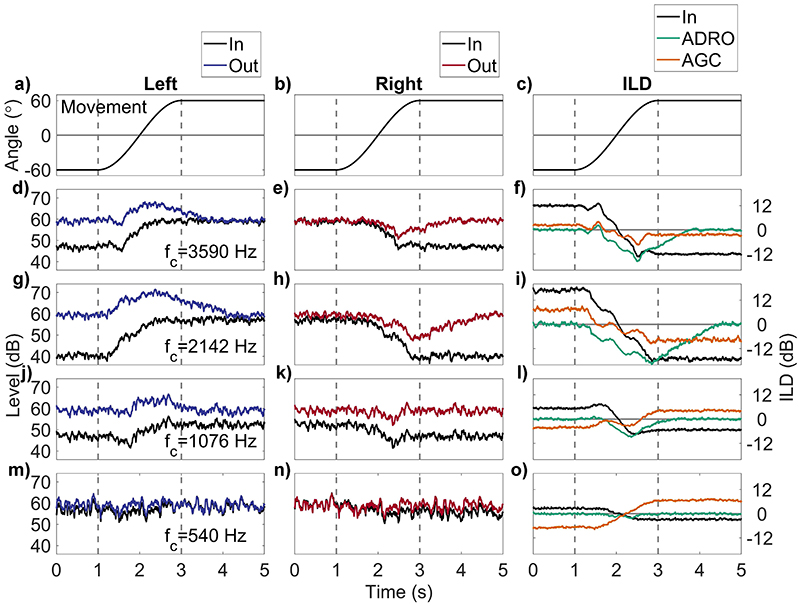
(Color online) Left and right ear inputs/ADRO™ output and ILD plots for four frequency channels, for a SSN input at 64 dB SPL (left ear). Rotational velocity is 60°s^−1^ from −60° to 60°. The left column (a, d, g, j, m) shows the inputs (black), and ADRO™ outputs (blue) at the left ear. The middle column (b, e, h, k, n) shows the inputs (black), and ADRO™ outputs (red) at the right ear. The right column (c, f, i, l, o) shows input (black), ADRO™ (green), and unlinked standard AGC (orange) output ILDs for four frequency channels. Standard AGC parameters are attack/release time 60/400 ms, compression ratio 12:1, and threshold 60 dB SPL. The rows show filter channels decreasing in frequency from top to bottom. f_c_ is the center frequency of the filter channel. The areas of the plots bounded by the dashed lines show the duration of rotational movement.

Figure 9 shows the left and right ear input and output levels for four frequency bands, for a two-second head movement from −60° to 60°, together with the resulting ILDs. Also, shown (in orange) are the ILDs for a standard AGC with attack/release times of 60/400 ms, a compression ratio of 12:1, and a threshold of 60 dB SPL. The top row [Figs. 9(a)–9(c)] shows the head movement (repeated for ease of reference), and the rows below show plots in order of decreasing channel frequency. The left column [Figs. 9(a), 9(d), 9(g), 9(j), and 9(m)] shows the input/output levels at the left ear, the middle column [Figs. 9(b), 9(e), 9(h), 9(k), and 9(n)] shows the input/output levels at the right ear, and right column [Figs. 9(c), 9(f), 9(i), 9(l), and 9(o)] shows the input/output and standard-AGC ILDs.

Parameter settings are identical in each channel for ease of comparison with respect to target output level, threshold, and maximum output level, and each frequency channel operates independently. The input level change during head movement differs across frequency, and this interacts with ADRO™ to result in different *durations* of output level change across frequency. Overshoot of up to 2 s (from 3 s onwards) occurs in both left and right ears [e.g., the middle row, Figs. 9(g)–9(i), in contrast to the unlinked standard AGC, which only showed overshoot in the ear that decreased in level. The static input is also whitened by the multichannel compression, and head movement reduces this whitening effect, as the gain applied to each channel is too slow to maintain the same level in each channel. The perceptual consequences of these differing durations of level change will be addressed in Sec. VII. The right column [Figs. 9(c), 9(f), 9(i), 9(l), and 9(o)] shows the input/output ILDs and the AGC output ILDs. Independent multichannel compression (green) leads to 0 dB ILD across frequencies prior to head movement, in contrast to the input ILDs that show the expected increasing ILD with frequency, and the standard AGC which shows the same range of ILDs as the input, shifted negatively by a combination of head-shadow, pre-emphasis, and compression. As the head moves, the input and standard AGC ILDs move towards 0 dB ILD and then change sign, whereas the ADRO™ output ILDs decrease from 0 at different rates in each frequency band, so that the input and ADRO™ ILDs are identical around the point where the input ILDs are at their minimum. The ADRO™ ILDs then increase back to 0 dB at similar rates in each channel, the duration of the increase and overshoot being dependent on the minimum ILD reached.

The ILD summary metrics, in this case applied to individual frequency bands, are shown in Table VI. The metrics show that overshoot duration increases with frequency, from 0s at 540 Hz, up to 1.3 s at 2.14 kHz, reducing to 0.7 s at 3.59 kHz. The initial difference follows the same pattern, increasing from 2.8 dB (540Hz) to 16.6 dB (2.14kHz), falling to 12.0 dB at 3.59 kHz. The trajectory error (from 3.0 to 17.3 to 11.7 dB for the same frequencies) and max deviation (from 2.5 to 19.6 to 15.6 dB) also follow the same pattern. It can be seen that ADRO™ significantly distorts the pattern of ILDs present in the input.

## VI XDP™ and VOICEGUARD™ SIMULATION AND RESULTS

### A Simulations

**Fig. 10:**
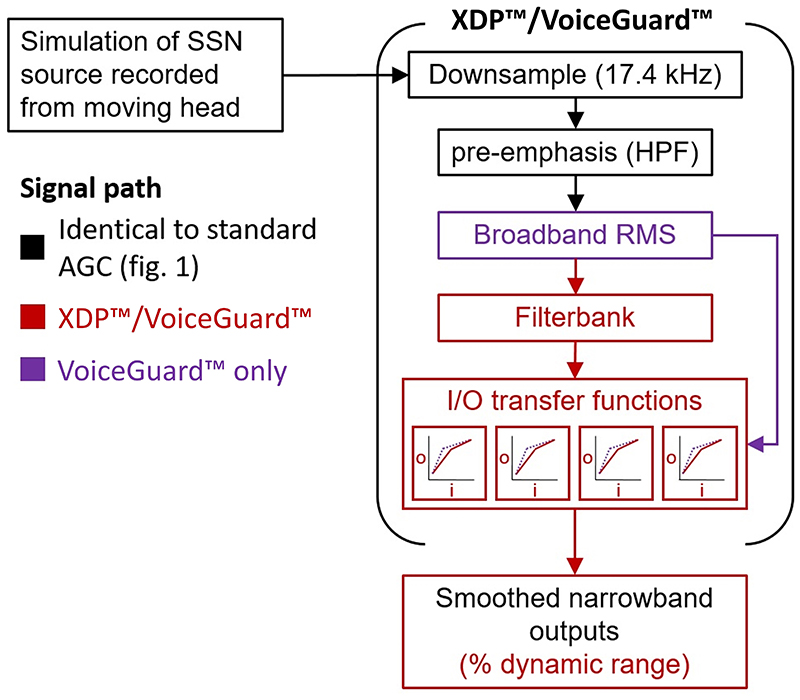
(Color online) Schematic showing the signal paths for the xDP™ and VoiceGuard™ simulators. Four I/O transfer functions are shown to reflect the four independent frequency channels in the systems.

VoiceGuard™ was developed by Oticon as an adaptive version of their previous xDP™ system, which could be described briefly as a four-channel compressor with an instantaneous attack and release time. The signal path is shown in Fig. 10. It is applied at the end of the signal chain, and converts input level in dB into percentage of electrical dynamic range *via* four independent input/output (I/O) transfer functions, across four frequency bands (center frequencies: 0.406, 1.125, 2.273, and 5.254kHz). The transfer functions are bilinear, defining two compression ratios and a threshold/kneepoint between the two. The output kneepoint is always set to 75% of the electrical dynamic range in linear *μC* of the corresponding band, and the input level at which this kneepoint occurs can be altered within each frequency band. In the previous xDP™ implementation, the kneepoints were manually selected by the clinician to optimise for a quiet, medium, or loud environment. In VoiceGuard™, the knee-points are automatically set based on the average level of sounds in the listener’s environment. The environmental level tracker has a time constant of 1.5 s, and the knee-points are selected in 3 dB steps. A hysteresis algorithm prevents any unwanted jitter between knee-points as the environmental level changes (Segovia-Martinez *et al*., 2016). When xDP™ or VoiceGuard™ are applied in CIs, the output levels are defined in terms of percentage of electrical dynamic range. The system converts input dB SPL directly to percentage of dynamic range. Therefore, the output knee-point is not 75 dB SPL, but 75% of dynamic range. Oticon maps their input/output function across a numerically equal range (i.e., the compression slope starts at 20 dB SPL = 20%, and ends at 95 dB SPL = 95%), so the output units are somewhat arbitrary.

### B Results

**Fig. 11:**
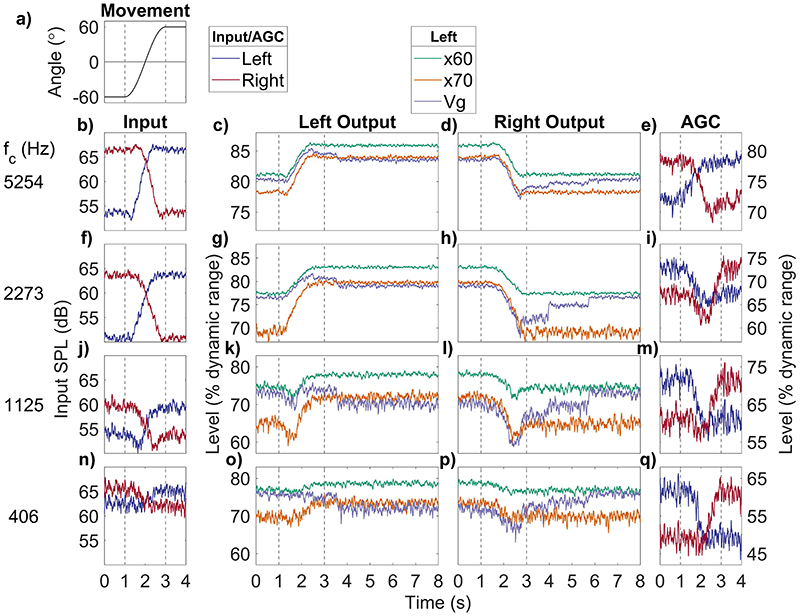
(Color online) Head movement, left and right level input, xDP™, VoiceGuard™, and single-channel AGC (for comparison) outputs for a SSN input at 64 dB SPL (left ear) for four frequency channels. The top left plot (a) shows head movement. The left column (b, f, j, n) shows input levels in each frequency band (high to low) in dB SPL (left, blue; right, red). The middle columns show the left (c, g, k, o) and right (d, h, l, p) output levels in percentage of dynamic range respectively. The right column (e, i, m, q) shows the left and right output levels for a single-channel AGC with attack/release time 60/ 400 ms, compression ratio 12:1, and threshold 60 dB SPL, converted into percentage of dynamic range assuming a 60 dB IDR and Advanced Bionics linear dB-to-% mapping (Vaerenberg *et al*., 2014). The bottom four rows (b–q) show frequency channels, decreasing from high to low. *f_c_* is the center frequency of each filter. “x60” (green) is the XDP™ output for an environmental level set to 60 dB SPL output, “x70” (orange) is the xDP™ output for a 70 dB SPL level, and “Vg” (purple) is the VoiceGuard™ output. Rotational velocity is 60°s^−1^ from −60° to 60°. The areas of the plots bounded by the dashed lines show the duration of rotational movement.

Figure 11 shows the input [left column, Figs. 11(b), 11(f), 11(j), and 11(n)] and output levels (middle columns, Figs. 11(c), 11(g), 11(k), 11(o), and 11(d), 11(h), 11(l), and 11(p)] at the left and right ears in four frequency channels (note that the frequency allocation is altered from the standard and ADRO™ systems described previously), presented in percentage of dynamic range. Three different Oticon systems are shown: the fixed knee-point xDP™ system assuming an environmental sound level of 60 dB SPL (x60, green) or 70 dB SPL (x70, orange), and the VoiceGuard™ system (purple), which automatically selects knee-points in each channel based on an environmental sound-level tracker. The response of a standard AGC is also shown by the blue and red lines in the fourth column. This response is also expressed as a percentage of dynamic range, assuming a 60 dB input dynamic range (as used in Advanced Bionics devices). As shown and discussed by Archer-Boyd and Carlyon (2019), the outputs for the lower three frequency channels and for the standard AGC show an ILD whose sign is opposite to that occurring at the input. These reversals arise because the compression in the standard AGC is driven by the highest-frequency channels, where the level changes are greatest, but applied equally to all channels, including those at low frequencies where the head movement does not strongly influence the input level at each ear.

For the fixed kneepoint xDP™ systems, the *reversal* of level changes seen in the standard AGC do not occur. This is because in these multi-channel systems the compression in each frequency band is driven by the input level in that band. Compression is greater using x60 than x70, as the input level is higher up the compressive function used. As the compression mapping has no attack and release time (or equivalent), the compression is effectively instantaneous, aside from the delay that all audio buffer-based signal processing produces. Therefore, the shape of the level change during head movement is not affected beyond compression (i.e., no overshoot or level change reversal). Therefore, xDP™ preserves the trajectory of the change in level at each ear across frequency bands, and lower kneepoints unsurprisingly lead to greater compression of the amount of change in level. The output of the VoiceGuard™ system (purple) is a little more complex. In static conditions, it places the output closer to the output kneepoint (at 75% of the electrical dynamic range in each channel) across all frequencies than either xDP™ setting. During head movement, input level changes are compressed at the output and may change abruptly by a few dB, as in the lowest frequency right output [Fig. 11(p)], for example. This is caused by a step change in knee-point used (and its corresponding input/ output mapping function), driven by the environmental level tracker that defines knee-points in 3 dB steps. After the head has stopped moving and the input levels stop changing, the output level continues to change up to 3 s after the head movement (from 3 s onwards). This occurs because, although the compression *per se* is instantaneous, the change in kneepoint is driven by the slow-acting broadband environment detector. These are step changes of up to 5 dB. Between the step changes, level is constant after the head movement stops.

**Fig. 12:**
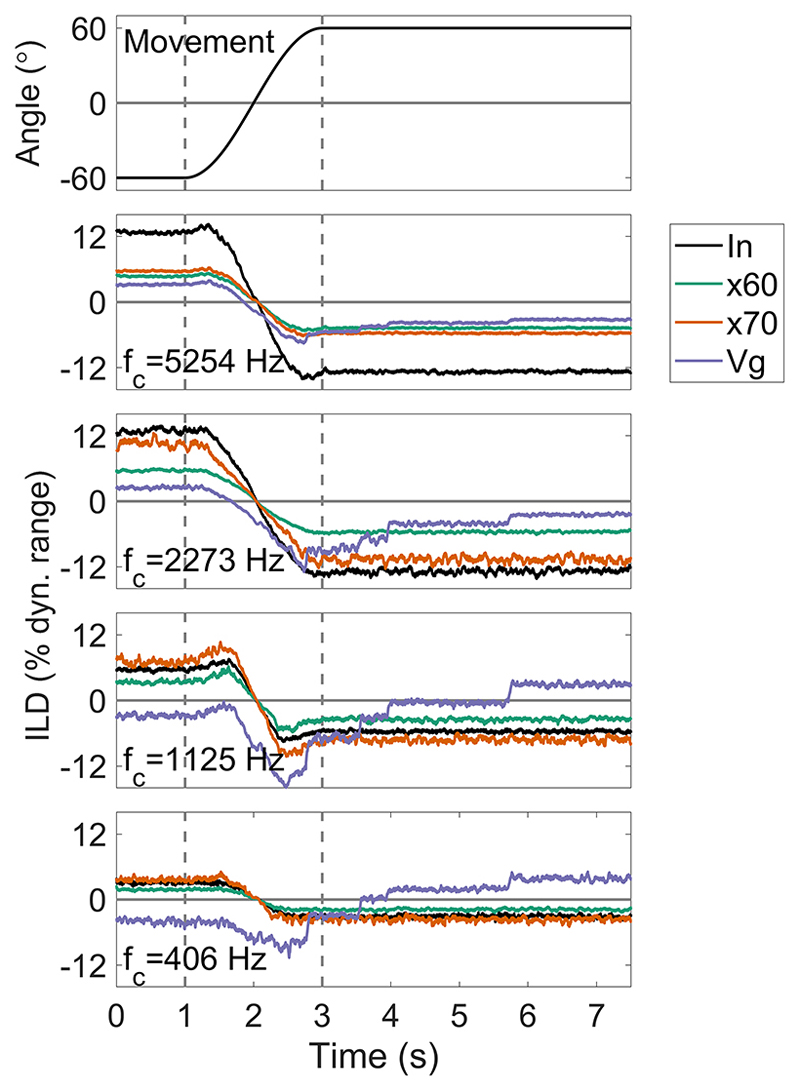
(Color online) Input xDP™ and VoiceGuard™ outputs ILDs for four frequency channels, for a SSN input at 64 dB SPL (left ear). Rotational velocity is 60°s^−1^ from −60° to 60°. The top plot shows the head movement. The lower plots show frequency channels, increasing from low to high. *f_c_* is the center frequency of each filter. “In” (black) is the input, “x60” (green) is the xDP™ output for an environmental level set to 60 dB SPL output, “x70” (orange) is the xDP™ output for a 70 dB SPL level, and “Vg” (purple) is the VoiceGuard™ output. *f_c_* is the center frequency of the filter channel. The areas of the plots bounded by the dashed lines show the duration of rotational movement.

Figure 12 shows the input, xDP™, and VoiceGuard™ outputs for four frequency bands, for a two-second head movement from −60° to 60°, based on the level changes seen in Fig. 11. The top plot shows the head movement, the lower plots show plots in order of decreasing channel center frequency.

It can be seen that the x60 (green) and x70 (orange) settings closely reproduce the variation in level with head position, and hence the ILDs, in the two lowest channels, and reduces the ILDs in the two highest channels. An exception to this general rule occurs in the 1125-Hz channel, where the x70 setting increases the ILD relative to the input. The VoiceGuard™ (purple) output ILDs show a reversal in sign across frequency compared to the input ILDs. This is similar to the ILD reversal across frequency that was shown in Fig. 9 (right column) and in Archer-Boyd and Carlyon (2019) for the standard unlinked AGC. This reversal occurs because instantaneous multi-channel input-output mapping functions are driven by a broadband environmental sound level tracker with a long time constant (1.5 s). The level tracker is very similar in functionality to the envelope tracker used in a single-channel unlinked standard AGC, and produces similar output behaviour when the head is static. When the head moves, the ILD change is compressed and mimics the input level change, unless a new knee-point is selected, as discussed in relation to Fig. 11. Again, step changes in ILD continue up to 3 s after the head has stopped moving (from 3 s in Fig. 12).

The summary metrics are shown in Table VII and some of them will now be highlighted. The metrics are calculated for four frequency bands, two xDP™ knee-point settings, and VoiceGuard™. Where required, the values have been expressed in dB rather than percentage of dynamic range in order to aid comparison with the values from other systems. Overshoot duration is 0s for xDP™ as the system has no time constant. The initial differences are a similar range across frequency for both the x60 (lower threshold, more compressive mapping) and x70 settings (0.8 to 4.8 dB and −0.3 to 4.2 dB, respectively). The values of the transfer function knee-point in relation to the input levels means that the x70 setting slightly enhances (equal to a negative initial difference) the input ILD in the lower two frequency bands, by 0.3 and 0.8 dB. Error trajectory increases with increasing frequency, and x70 achieves a lower error than x60 (0.5 to 5.4 dB, and 0.9 to 6.1 Db, respectively). Max deviation varies between 0.3 and 2.2 dB, with little apparent relationship with frequency.

The three lowest frequency bands in the VoiceGuard™ produce an overshoot value of 2.73(1) s, whereas the highest frequency overshoot duration is 0.96 s. This is because the time constant is the same for each frequency band, but the highest frequency input is mapped to the compressive part of the input-output function, resulting in a smaller step-size at the output. This results in the output ILD being within 0.5 dB of the final ILD value sooner than in the other frequency bands. Initial differences are larger, and follow a similar (though smaller in extent) pattern to ADRO™, increasing from 4.2 dB at 0.406 kHz to 6.3 dB at 2.273 kHz, and reducing to 5.7 dB at 5.254 kHz. Error trajectory follows a similar pattern, increasing from 5.1 to 8.7 dB, then reducing to 7.7 dB at the highest frequency. The largest max deviation occurs at 1.125 kHz (11.3 dB), and the smallest is at the highest frequency (2.6 dB).

The summary metrics suggest that VoiceGuard™ distorts output ILDs more than xDP™, and that inputs that crossover the compression thresholds of the input-output functions result in small enhancements increases in output ILD relative to the input ILD.

## VII Discussion

We have extended the simulations and analyses presented by Archer-Boyd and Carlyon (2019) to investigate the effect of altering parameters in different implementations of AGCs on the unilateral and bilateral output of CIs during head movement. A perfect AGC system would provide audibility, improve speech intelligibility, and preserve static and dynamic ILD cues across frequency. The limited dynamic range of hearing of CI listeners means that some kind of AGC is required to compress the input dynamic range, which will inevitably result in a trade-off between audibility, intelligibility, and localization cues (in the case of CI users we consider only ILDs). Here, we have considered four approaches to AGCs available in CIs: unlinked and linked AGC, ADRO™, xDP™, and VoiceGuard™. Each of these approaches degrades ILDs cues in at least one way, and we discuss each of these limitations in turn below. The relative advantages and disadvantages of each system are summarized in Table VIII.

### A Distortions produced by AGC systems

#### 1 Overshoot

The overshoot effect due to AGC has been shown for level changes in static sources (Stone *et al*., 1999) and for head movement (Brimijoin *et al*., 2017; Archer-Boyd and Carlyon, 2019). Here, the contribution of different parameters to the degree of overshoot has been investigated. The main contributing parameter in standard broadband AGCs was the release time, with some contribution from the compression ratio used. Release time had the largest effect partially because it is the value that changes the most across clinical devices [bottom row, Figs. 5(p)–5(r)]. Attack and release time settings chosen conservatively (longer attack and release times) to maintain both a similar level at all times and less envelope distortion will necessarily produce greater over-shoot than faster, less conservative settings that protect listeners from sudden high level sounds at the possible expense of distorting the speech envelope. Linked broadband AGC did not produce overshoot due to head movement because overshoot is produced by the side that initially decreases in level (Fig. 7). At the time-point where overshoot occurs, this side has the least attenuation applied to it of the two, and the linked AGC only uses the maximum attenuation value from either side of the head. An unlinked multi-channel system, such as ADRO™, produces variable overshoot across frequencies [right column, Figs. 9(c), 9(f), 9(i), 9(l), and 9(o)]. This is because the level change due to head movement is greater at high frequencies than at low, and the gain change rate is the same in each channel. Therefore, larger level changes result in more overshoot. The XDP™system does not overshoot simply because it has no time constant to cause one (Fig. 12). However, the long time-constant used by the VoiceGuard™ system to control the instantaneous mapping does produce overshoot because VoiceGuard™ uses a broadband control signal; in this case, the duration of the overshoot is similar across frequencies (Fig. 12). It essentially introduces a release time to xDP™. VoiceGuard™ also changes the knee-points of the instantaneous mapping in discrete 3 dB steps, which means that the overshoot is also stepped. The kneepoints are different for some frequency bands, so there is a variation in overshoot duration across channels that is much smaller than that seen using ADRO™.

#### 2 ILD reversal at low frequencies

The reversal of ILDs across frequency due to unlinked broadband AGC has been reported previously (Dorman *et al*., 2014; Archer-Boyd and Carlyon, 2019). Here, it has been shown that the degree of reversal is dependent on the compression ratio used (Fig. 6). It occurs using broadband unlinked AGCs because the amount of attenuation applied to the signal is determined by its overall level, and the difference in level between the ipsilateral and contralateral ears is greatest at higher frequencies. This difference is enhanced by the high-pass pre-emphasis filter and fixed makeup gain. Therefore, low- and high-frequency ILDs are reduced by the same amount, but the smaller low-frequency ILDs become negative at sufficiently high compression ratios. Large ILDs at low frequencies can occur naturally for sound sources that are close to the head, but these are coupled with larger ILDs at high frequencies (Brungart and Rabinowitz, 1999). When head movement is added to this scenario, the negative low-frequency ILDs increase and the positive high-frequency ILDs decrease so that at some point in the movement, the broadband output ILD is briefly identical to the broadband input ILD. At the end of the symmetric movement (and after any time period of overshoot), the ILDs across frequency are reversed. It is not yet known how this would be perceived by listeners (see Sec. VIIC). However, it may be heard as a timbral change rather than a location change, depending on what frequencies the listener is using to attempt localization of a source (Archer-Boyd and Carlyon, 2019). Depending on the duration of overshoot, the timbral change may continue after head movement has stopped. In the linked broadband case, ILDs are preserved, and no ILD reversal across frequency occurs. However, during head movement, the change in ILD is driven largely by sequential monaural changes, not simultaneous binaural changes. Initially the contralateral side increases in level as the head approaches 0°, then the other ear decreases in level as the head moves away from 0°. It is unclear how this would be perceived by listeners. Multichannel systems like ADRO™ do not cause ILD reversal at low frequencies, as each channel is independent. However, they can produce 0 dB ILDs across frequency frequencies [right column, Figs. 9(c), 9(f), 9(i), 9(l), and 9(o)]. This is because independent frequency channels on the left and right side of the head are unlinked and programmed to achieve the same output level. When the head moves, absolute ILDs increase across frequency, but they are initially of the opposite sign to the natural ILDs, much like the standard unlinked broadband AGC. However, unlike the standard unlinked broadband AGC, the ILDs across frequency are at their most different at 0°, and all frequency bands return to 0dB after the head has stopped moving, albeit with different durations of overshoot as previously discussed. The multi-channel, instantaneous xDP™ system does not cause ILD reversal at low frequencies for two reasons (Fig. 12). First, the compressive function used does not depend on input level, so each device is not attempting to output signals at the same level. Second, the input is split into several frequency bands, and therefore the attenuation of low frequencies is not dependent on the level of high frequencies, which is a major cause of ILD reversal at low frequencies in the unlinked broadband AGC. VoiceGuard™ reintroduces a slow time-constant control signal to an instantaneous multiband compression system, which results in ILD reversal at low frequencies similar to the unlinked broadband AGC case, since the control signal used is also broadband (Fig. 12).

#### 3 Comfortable listening level and intelligibility

A comfortable listening level is important for a CI user’s listening experience. The systems simulated here fall into two main groups. The unlinked broadband AGCs maintain a comfortable listening level by keeping the overall broadband output level for supra-threshold input sounds around the compression threshold at both ears. The compression ratio is the most important parameter for controlling how much the level is allowed to vary, and spectral shape of the input is not altered by the compressor. The methods used for increasing the level of very low level inputs (e.g., Cochlear’s “Whisper™”) have not been considered here. Linked broadband AGC also does not alter spectral shape. However, the contralateral ear is lower in level than the ipsilateral (Fig. 7). This output ILD may be larger than the input ILD, and reduce the level of the source in the contralateral ear. This occurs because the same attenuation is applied to both sides to preserve ILD. However, the preserved ILD is larger than the natural ILD due to the use of a pre-emphasis filter giving more weight to higher frequencies. A high compression ratio also exacerbates this effect, as it increases the attenuation applied at both ears.

Multichannel systems like ADRO™ maintain comfortable listening levels in each channel. By design, these systems alter the spectral shape of the input to fit the dynamic range of the listener. The whitened signal may technically reduce spectral contrast; however, it will set the output level closer to a comfortable listening level across frequency. The variation of ILDs across frequency will not be preserved [right column, Figs. 9(c), 9(f), 9(i), 9(l), and 9(o)]. The multichannel xDP™ system similarly whitens the input to maintain comfortable listening levels (Fig. 11). However, because the input-output function is fixed, and the number of channels is much fewer than ADRO™, the spectral whitening is reduced relative to ADRO™. The amount of whitening produced by the VoiceGuard™ system depends on the input level and spectrum, as the broadband control signal is designed to keep the compressive knee-point closer to the level of the input sound (Fig. 11). Therefore, a high level input would be compressed and whitened less using VoiceGuard™ than xDP™, and a low level input would be compressed and whitened more.

#### 4 Distortion of fast envelope fluctuations

The time constants used clinically by CIs are generally slow enough to preserve fast envelope fluctuations, and indeed given CI listeners’ dependence on the temporal envelope of speech, the use of slow time constants is not surprising. There are two exceptions to this when considering standard broadband AGCs.

The first is the fast-acting transient compressor used by AB and Med-El in their dual-loop AGCs. The second is the Cochlear compression limiter. Both systems are used to protect listeners from high-level sounds. Both systems have short attack and release times and high compression ratios and relatively high thresholds. However, the fast-acting part of the dual-loop AGC is only applied if the ongoing level of the signal changes rapidly by more than 8 dB from its previous level (i.e., it detects and limits loud transients). Therefore, the dual-loop system will only distort fast speech envelope fluctuations if a loud transient is also present. The Cochlear limiter on the other hand is always on and will distort any sound above the adjustable threshold, speech, transient, or otherwise. These distortions have been shown to harm speech intelligibility (Khing *et al*., 2013).

The multi-channel ADRO™ system does not use a compression ratio, and the gain changes in each channel are slow (<10 dBs^−1^), meaning that fast temporal envelope fluctuations are preserved, and theoretically, audibility is maintained. Despite the spectral whitening that ADRO™ produces, several studies have shown that ADRO™ improves speech intelligibility (e.g., James *et al*., 2002). This suggests that spectral whitening does not have a strong detrimental effect on intelligibility in the scenarios tested.

The xDP™ and VoiceGuard™ systems are essentially an instantaneous multi-channel compressor, meaning that fast temporal envelope distortions are likely. However, the compressive part of the input-output function is low in ratio, between approximately 1.5 and 3:1 depending on the kneepoint value, and the function is fixed. Therefore, the input level where maximum fast temporal envelope fluctuations would occur is around the knee-point. The VoiceGuard™ system makes temporal distortions more likely as it adaptively shifts the kneepoint based on the broadband sound level. However, despite these theoretical issues, XDP™ has been shown to improve speech intelligibility when compared to standard broadband AGCs (Bozorg-Grayeli *et al*., 2016).

#### 5 Summary

As previously stated in this and other studies (e.g., Wiggins and Seeber, 2011; Dorman *et al*., 2014), the use of compression results in a trade-off between audibility and the preservation of ILDs, especially dynamic ILDs produced by head movement. The results of the dynamic simulations presented here and previous work focussing on speech intelligibility have been considered. The summary metrics used to quantify the degree of ILD distortion in a comparable way allow theoretical observations to be made in the absence of human perceptual data. For the standard, unlinked AGC, changing the attack time did little to alter the trajectory error (Table II). Increasing the release time had a variable effect on trajectory error (Table III). This occurred because a slow release time means that the output ILD trajectory is more similar to the input ILD (once initially aligned). However, a slow release time greatly increased the duration of the overshoot, and the trajectory error included this portion of the output ILD. Since the duration of head movement and overshoot varied, the variable trajectory error showed the complex interaction between them. Though the largest effect of the compression ratio appeared to be the start and end output ILDs, the trajectory error also increased with increasing ratio, increasing with the max deviation value (Table IV). In this case, these two metrics, trajectory error and max deviation, described the simpler output ILD trajectory and showed that it was closer to the input ILD trajectory. The linked AGC had a similar trajectory error to the unlinked. However, the initial difference values had opposite signs, showing that the type of ILD trajectory distortion introduced by the system was different, and due to effects of compression when the head was static (Table V). Finally, the summary metrics showed the similarities between the ADRO™ and VoiceGuard™ responses (Tables VI and VII). Both produced their largest trajectory errors when the input ILD was largest, and all the metrics were proportional to one another. The xDP™ showed very little distortion of the ILD trajectory at the output in some cases, and this could be observed clearly in the summary metrics as well as in the plots (Table VII, and Figs. 11 and 12).

It appears that the systems that provide the best trade-off are a standard, unlinked AGC with a relatively fast release time and a low compression ratio, similar to AGC used in the Med-El device, or the instantaneous input-output mapping of the xDP™ system. At the output of the standard AGC, these parameter settings preserve the sign of broad-band ILDs when the head is static, and the shape of the broadband change in ILD during (and immediately after) head movement. Monaural level changes across frequency are also preserved (discounting the effect of pre-emphasis), as are fast envelope fluctuations. ILDs are compressed in a constant, predictable way, and given the reduced dynamic range of CI listeners, this may be something that can be learned by the listener. A disadvantage is that the sign of the ILD in low-frequency channels can be opposite to that at the input, and opposite to that in the higher-frequency channels, potentially leading to a blurred spatial image (Wiggins and Seeber, 2011). This does not occur in the xDP™ system. However, the major drawback of this system is that it does not account for the relative magnitudes of the input ILDs across frequency, and whitens the input spectrum.

### B Head movement and dynamic ILDs

In a previous study (Archer-Boyd and Carlyon, 2019), we modeled the effect of linear head movements on dynamic ILDs. The present study instead modeled sinusoidal head movements, which better mimic real movements (e.g., Brimijoin *et al*., 2013). This resulted in two changes from the previous study. First, there were time periods at the beginning and end of each movement (seen most clearly in the 30°s^−1^ condition) where there was little to no change in the natural level/ILD. This is due to the interaction between the position of the devices on the head, the start and end positions of the head, and the sinusoidal movement trajectories. For the impulse responses used (taken from behind-the-ear hearing aids), there is little change in natural ILD around ±60°, and as angle increases, ILD change naturally decreases. This, combined with a low velocity, resulted in longer time periods of little to no change in level/ILD. Second, velocity at the mid-point of the head movements was higher than the average over the whole movement and therefore higher than they were in the previous paper (Archer-Boyd and Carlyon, 2019).

Natural ILDs mostly increase in magnitude with increasing absolute angle from 0° (source in front of the listener’s head) (Macaulay *et al*., 2010). Natural ILDs also increase with frequency (Blauert, 1997). The simulated head movements from −30° to+30° produced a change in natural ILDs from positive to negative values. Rate of change was obviously smaller at lower than at higher frequencies, and varied during the movement due to the sinusoidal head movement used, and diffraction around the head and torso (pinna filtering was not a factor, as the microphones were placed above the pinna).

Real-world dynamic ILDs rarely occur in the absence of any other changes in the source signal. Indeed, the advantage of simulating the AGC output is that parameters can be examined in isolation before considering their relative contributions to perception. The angular range of the head movements considered here is approaching the maximum that a human may be expected to make at velocities that are plausible (Hadar *et al*., 1983; Hadar *et al*., 1985). In the real-world, the magnitude of ILD changes due to head (or source) movement would be comparable to the difference in overall ILD between a low and high frequency sound in the far-field (>1m) (Blauert, 1997), or a sound source moving radially towards or away from the head in the near-field (<1 m) (Brungart and Simpson, 2002). Envelope changes in the signal (e.g., speech) would complicate interpretations further, though there is evidence that relatively low compression ratios (3:1) affect the lateral position of speech from static sources (Wiggins and Seeber, 2011), so the effect discussed here would be expected to impact the perception of dynamic ILDs.

Finally, histograms of ILD distributions for static speech sources over time have been shown to widen with increasing reverberation, which would make distortions to dynamic ILDs more difficult to detect clearly (Catic *et al*., 2013). In short, there are many factors involved in examining dynamic ILDs, and our simulations do not consider them all in order to allow clear interpretations of dynamic ILDs produced.

### C Future work and limitations

The simulation used in this study did not include the mapping of the level in each channel post-audio processing to the stimulation current of each electrode that is used in CIs. In Advanced Bionics devices, this is another free parameter (the so-called “maplaw”) that can affect level and ILD perception. These mappings were not included in this simulation as they are highly dependent on the user’s preference and fitting, and do not change dynamically with level.

Another factor affecting the dynamic ILDs presented to the CI listener is the coding strategy of the device. Continuous interleaved sampling (CIS) sends pulses to every electrode on every sweep of the array, whereas so-called “N-of-M” strategies choose N spectral peaks in each audio frame, and send pulses to the electrodes matched to the frequencies of those peaks, from a possible M electrodes. Using a CIS strategy, the broadband ILDs will be presented as shown here, whereas an N-of-M strategy, picking peaks independently at each ear, will alter both the spectral balance of the presented ILDs, and the overall, broadband ILD. An algorithm that synchronized the N-of-M strategies between the ears to preserve ILD cues was recently presented by Dennison *et al*. (2019), but did not significantly improve localization or motion perception accuracy in bilateral listeners.

Some elements of the CI system simulations have not been included here, as they deal with types of signal that we have not used. These include low input-level processing such as Whisper™ from Cochlear, the fast-acting compressor that is used in the standard AGC (found in AB and Med-El devices) to suppress sudden, loud transient sounds, and Oticon’s multichannel noise-reduction algorithm (known as “Voicetrack”). Future work will investigate the effect of multiple speech sources at different levels on dynamic ILDs, and will therefore include these algorithms in simulations. In addition, more advanced forms of linked AGC available in the literature (but not clinically) have not been considered. These include a linked medial olivocochlear-inspired system (Lopez-Poveda *et al*., 2019; Lopez-Poveda *et al*., 2020), the linked single-channel system used by Potts *et al*. (2019), and the linked and multichannel system by Spencer *et al*. (2019). In all cases, speech intelligibility was improved for some sets of parameters, as was localization with a stationary listener and source where tested. Future work will investigate the effect of head movement or dynamic sources on these systems.

Finally, these simulations use an anechoic SSN as an input. This means that our results are more easily compared to both the previous paper and earlier work on the effect of compression on ILDs. SSN has the same long-term spectral content as speech, but the amplitude modulation is not the same. Adding reverberation would reduce the modulation depth of the speech, will resulting in a greater spread of ILDs due to reflections. The interaction between these parameters and compression may be a topic for future study.

## VIII Summary and Conclusions

A simulation of bilateral CI pre-processing and several different AGCs on a rotating head was used to investigate the effect of changing AGC parameters and systems on output ILDs during head movement. Changing the release times and compression ratios (within clinically plausible limits) had the largest effect on output ILDs during head movement in single-channel AGCs. Linking AGCs preserved dynamic ILDs at the expense of the trajectory of monaural level cues and level at the contralateral ear. Multichannel alternatives to AGC, such as ADRO™, had the effect of whitening the output spectrum and causing ILDs to change at different rates and for different durations in each frequency channel. Instantaneous post-processing compression, as used by Oticon, preserved ILD cues and reduced the effect of headshadow. Adding a slow-acting control signal to this instantaneous compression resulted in similar behaviour to a singlechannel AGC.

A recommendation for the AGC type and parameters that would provide the best compromise between speech intelligibility and (static or dynamic) ILD cues is not possible in this study as intelligibility was not measured. However, what can be concluded from this study is that the longer the time-constants used, the greater the distortion to dynamic ILDs. In addition, fully independent channels in multi-channel systems such as ADRO™ can distort dynamic ILDs still further. If instantaneous compression is not possible or preferable, then release times or time constants of approximately 0.5 s, and compression ratios towards the maximum found in hearing aids (e.g., 3:1) represent the best settings for dynamic ILD preservation.

## Supplementary Material

Appendix

## Figures and Tables

**Table I:** The ANSI-defined attack and release times, compression ratios used here, and the time constants used to obtain them. The methods used to relate the ANSI times to the time constants are given in the Appendix.

ANSI attack time (ms)	ANSI release time (ms)	Compression ratio	Attack time constant (ms)	Release time constant (ms)
60	400	3:1	41	347
60	400	12:1	31	305
60	400	∞(1 000 000:1)	29	296
60	100	12:1	31	77
60	400	12:1	31	305
60	1600	12:1	31	1214
15	400	12:1	11	305
60	400	12:1	31	305
240	400	12:1	107	305

**Table II:** The effect of changing the attack time on the summary metrics. The metrics are explained in Sec. IIC.

Attack time (ms)	Head velocity (degrees/s)	Overshoot duration (s)	Trajectory error (dB)	Initial difference (dB)	Max deviation (dB)
15	30	0.00	6.41	5.32	1.84
60	30	0.00	6.43	5.38	2.25
240	30	0.00	6.19	5.38	2.32
15	60	0.00	5.56	5.39	3.28
60	60	0.00	5.36	5.37	3.19
240	60	0.00	5.16	5.28	4.13
15	120	0.26	5.77	5.40	4.93
60	120	0.28	5.60	5.38	5.11
240	120	0.29	5.29	5.55	6.29

**Table III:** The effect of changing the release time on the summary metrics. The metrics are explained in Sec. II C.

Release time (ms)	Head velocity (degrees/s)	Overshoot duration (s)	Trajectory error (dB)	Initial difference (dB)	Max deviation (dB)
100	30	0.00	6.97	5.45	0.73
400	30	0.00	6.43	5.38	2.25
1600	30	0.27	5.00	5.53	4.98
100	60	0.00	6.86	5.49	1.03
400	60	0.00	5.36	5.37	3.19
1600	60	1.52	5.87	5.14	6.81
100	120	0.00	5.85	5.35	2.10
400	120	0.28	5.60	5.38	5.11
1600	120	1.62	6.14	5.48	8.63

**Table IV:** The effect of changing the compression ratio on the summary metrics. These metrics are explained in Sec. II C.

Compression ratio	Head velocity (degrees/s)	Overshoot duration (s)	Trajectory error (dB)	Initial difference (dB)	Max deviation (dB)
3	30	0.00	3.14	2.79	1.42
12	30	0.00	6.43	5.38	2.25
∞	30	0.00	7.44	6.28	2.10
3	60	0.00	2.35	2.78	2.82
12	60	0.00	5.36	5.37	3.19
∞	60	0.00	6.67	6.61	3.69
3	120	0.24	2.22	2.81	4.10
12	120	0.28	5.60	5.38	5.11
∞	120	0.22	6.47	6.28	5.82

**Table V:** The effect of linking compressors on the summary metrics. The metrics are explained in Sec. II C.

	Head velocity (degrees/s)	Overshoot duration (s)	Trajectory error (dB)	Initial difference (dB)	Max deviation (dB)
**Linked**	60	0.00	5.28	–4.29	1.45
**Unlinked**	60	0.00	5.37	5.35	3.56

**Table VI:** Summary metrics across frequency using ADRO™. The metrics are explained in Sec. II C.

Centre frequency (kHz)	Head velocity (degrees/s)	Overshoot duration (s)	Trajectory error (dB)	Initial difference (dB)	Max deviation (dB)
0.54	60	0.03	2.97	2.76	2.52
1.076	60	0.22	5.00	5.34	8.90
2.142	60	1.35	17.29	16.61	19.62
3.59	60	0.67	11.69	11.97	15.56

**Table VII:** Summary metrics across frequency using Oticon xDP™60, xDP™70, and VoiceGuard™. The metrics are explained in Sec. II C.

Oticon	Centre frequency (kHz)	Head velocity (degrees/s)	Overshoot duration (s)	Trajectory error (dB)	Initial difference (dB)	Max deviation (dB)
xDP™ (x60)	0.406	60	0.00	0.93	0.78	2.21
	1.123	60	0.00	1.73	1.36	1.40
	2.273	60	0.00	5.36	4.34	0.32
	5.254	60	0.00	6.10	4.80	0.34
xDP™ (x70)						
	0.406	60	0.00	0.50	–0.32	0.83
	1.123	60	0.00	1.40	–0.82	1.93
	2.273	60	0.00	2.04	1.37	1.22
	5.254	60	0.00	5.35	4.22	0.34
VoiceGuard™						
	0.406	60	2.73	5.11	4.23	8.71
	1.123	60	2.75	5.68	5.06	11.32
	2.273	60	2.72	8.75	6.26	6.32
	5.254	60	0.96	7.66	5.69	2.60

**Table VIII:** Pros and cons of AGC and AGC-like systems. Positive effects are in **bold** and negative effects are in *italics*. Overshoot, Output ILD changes after input ILD stops changing; LF ILD reversal, ILD reversal at low frequencies; Presentation level, level of output; Whitening, spectral flattening; Fast env. distortion, distortion of fast changing envelopes (such as speech).

	Single, unlinked, 3:1		Linked Long release	Multi-channel		
	Release < 400 ms	Long release		ADRO™	xDP™	VoiceGuard™
Overshoot	**No**	*Yes*	**No**	*Yes*	**No**	*Yes*
LF ILD Reversal	*Yes*	*Yes*	**No**	**No**	**No**	*Yes*
Output level	Broadband	Broadband	*Reduced contralateral*	Per-channel	Per-channel	Per-channel
Whitening	**No**	**No**	**No**	*Yes*	*Yes*	*Yes*
Fast env. distort	**No**	**No**	**No**	**No**	*Yes*	*Yes*
