# Supplementary material for: Further simulations of the effect of cochlear-implant pre-processing and head movement on interaural level differences
Source: J Acoust Soc Am. Author manuscript; Available in PMC 2022 Jul 28. (PMC7613192; doi:10.1121/10.0005647)
Supplement: Appendix [file EMS151050-supplement-Appendix.pdf]

https://doi.org/10.1121/10.0005647

APPENDIX: A NOTE ON DESCRIBING DYNAMIC RANGE COMPRESSION

The definition of the envelope tracker given in Eq. (1) in the methods section produces output level behaviour similar to Fig. 4.5 from p. 104 of Launer *et al*. (2016), and crucially provides the best (though not an exact) fit to the output response of the slow-acting compressor in the AB BEPS+ simulator, which is based on the dual-loop compressor (e.g., Stone *et al*., 1999; Boyle *et al*., 2009). This definition is also similar to that used in the master hearing aid (Grimm *et al*., 2006). However, p. 233 of Kates (2008) defines the second line of Eq. (1) (containing β) as $\beta d[n-1]$, which results in a more linear change in dB when the compressor releases, instead of the gradual decrease in the rate of dB change seen in the simulation using Eq. (1). However, both definitions are correct, the definition used in Eq. (1) and in the simulations presented converge toward the input level, x, instead of 0, and as x is defined logarithmically in decibels and not linear units, this definition is used. In practice, the two definitions result in different release time constants for the same ANSI-defined release time, due to differences in the shape of the envelope tracker response to a stepped change in the input level.
